# Glenoid morphology in light of anatomical and reverse total shoulder arthroplasty: a dissection- and 3D-CT-based study in male and female body donors

**DOI:** 10.1186/s12891-016-1373-4

**Published:** 2017-01-10

**Authors:** Sandra Mathews, Marco Burkhard, Nabil Serrano, Karl Link, Martin Häusler, Nakita Frater, Ingeborg Franke, Helena Bischofberger, Florian M. Buck, Dominic Gascho, Michael Thali, Steffen Serowy, Magdalena Müller-Gerbl, Gareth Harper, Ford Qureshi, Thomas Böni, Hans-Rudolf Bloch, Oliver Ullrich, Frank-Jakobus Rühli, Elisabeth Eppler

**Affiliations:** 1 Institute of Evolutionary Medicine (IEM), University of Zurich, Winterthurerstrasse 190, CH-8057 Zurich, Switzerland; 2 Division of Gross Anatomy, Institute of Anatomy, University of Zurich, Zurich, Switzerland; 3 Medical Radiology Institute, Schulthess Clinic, Zurich, Switzerland; 4 Medical Faculty, University of Zurich, Zurich, Switzerland; 5 Institute of Forensic Medicine, University of Zurich, Zurich, Switzerland; 6 Institute of Neuroradiology, University Hospital Magdeburg, Magdeburg, Germany; 7 Musculoskeletal Research Unit, Department of Biomedicine, University of Basel, Basel, Switzerland; 8 Shoulder Unit, Queen Alexandra Hospital, Portsmouth, UK; 9 Shoulder Unit, Doncaster Royal Infirmary, Doncaster, UK; 10 Technical Orthopedics Unit, University Hospital Balgrist, Zurich, Switzerland; 11 Shoulder Surgery Unit, Ospedale Civico, Lugano, Switzerland

**Keywords:** Anatomical and reverse total shoulder arthroplasty, Suprascapular nerve, Screw placement, Glenoid cavity, Anteversion angle, Retroversion angle, Inclination angle, Dissection study

## Abstract

**Background:**

Placement of the glenoid baseplate is of paramount importance for the outcome of anatomical and reverse total shoulder arthroplasty. However, the database around glenoid size is poor, particularly regarding small scapulae, for example, in women and smaller individuals, and is derived from different methodological approaches. In this multimodality cadaver study, we systematically examined the glenoid using morphological and 3D-CT measurements.

**Methods:**

Measurements of the glenoid and drill hole tunnel length for superior baseplate screw placement were recorded to define size of the glenoid and the distance to the scapular notch on cadaveric specimens. Glenoid angles were determined on both, 3D-CT-scans of the thoraxes using the Friedman method and on subsequently isolated scapulae from 18 male and female donors (average 84 years, range 60–98 years).

**Results:**

Mean glenoid height was 36.6 mm ± 3.6, and width 27.8 mm ± 3.1 with a significant sex dimorphism (*p* ≤ 0.001): in males, glenoid height 39.5 mm ± 3.5, and width 30.3 mm ± 3.3, and in females, glenoid height 34.8 mm ± 2.2, and width 26.2 mm ± 1.6. The average distance from the superior screw entry to its exit in the scapular notch measured by calliper was 27.2 mm ± 6.0 with a sex difference: in males, 29.4 mm ± 5.7, and in females, 25.8 mm ± 5.9 mm with a minimum recorded distance of 15 mm. Measured by CT, the mean inclination angle for male and female donors combined was 13.0° ± 7.0, and the ante-/retroversion angle −1.0° ± 4.0°.

**Conclusion:**

This study is one of the first to combine dissection, including drill holes, with anatomical measurements and radiological data. In some women and smaller individuals, smaller baseplates should be selected. The published safe zone of 20 mm is generally feasible for superior screw placement, however, in small patients this distance may be substantially shorter than expected and start as of 13 and 15 mm, respectively. No correlation between glenoid height or width with the length of our drilling canal towards the scapular notch was found. Preoperative CT-based treatment planning to determine version and inclination angles is recommended.

## Background

Anatomical (TSA) and reverse total shoulder arthroplasty (RTSA) are effective treatment options for multiple disorders of the shoulder [1–4]. For long-term successful management, optimal positioning of the components is crucial [2, 5]. Implanting the glenoid component in an anatomical position is a challenge, which may explain the high rates of glenoid component loosening. This has been ascribed to limited bone stock of the available glenoid, the lack of reliable landmarks to determine the position of the blade of the scapula intra-operatively, and the poor understanding of the anatomical position, which shows great patient-specific variability. Surgeons may tend to aim for a ‘standard’ position of so-called neutral orientation of the glenoid component [6, 7].

Thus, orthopaedic surgeons emphasize a need for better understanding of the glenoid morphology to ensure proper sizing and correct placement of prosthetic components [8]. In particular, the consideration of glenoid size is of high relevance for long-term osseous integration [2, 9, 10]. Recent findings showed that small baseplates improve primary stability of the glenoid component in small glenoids [9], but published data for small glenoids for example in women are limited.

Furthermore, choice of inclination, version and rotation of the prosthetic glenoid component is mandatory for stability, a painless range of motion and to prevent impingement [5, 6, 11]. Thus, preoperative CT-based investigation is increasingly used to optimize pre-operative planning [12–15], but may not be easily accessible in all instances.

Nerve injury after RTSA has been reported with an incidence of 0.5-2.9% depending on the surgical approach [1, 16]. For implanting the glenoid component, there is a specific concern for the suprascapular nerve as it travels through the suprascapular notch where it is vulnerable to damage by the superior baseplate screw [17, 18]. Functional consequences of suprascapular nerve injury may be considered as minor since patients undergoing RTSA reportedly already have little function of the rotator cuff [19], but chronic pain and weakness may necessitate further medical intervention [18, 20, 21]. Furthermore, in patients with an intact rotator cuff, favourable influence of the rotator cuff has been reported for the range of motion and dynamic stabilisation of the anatomic and reverse total shoulder prosthesis [22, 23].

Data from human cadavers and/or scapular bones are essential to enhance knowledge of the glenoid anatomy in a representative elderly male and female population. Some studies have been conducted using isolated scapular bones [24–32], and anatomical studies are excellent tools to investigate safe drilling distances. However, such studies have been mainly directed to the posterior aspect of the scapula to optimise interventions such as the Bankart and Latarjet procedures and rotator cuff repair [2, 27, 33–40].

In a recent study on the Latarjet procedure [2], a safe zone for placement of graft-fixing screws was proposed as an approximately 2 cm-wide area medial to the glenoid rim, similar to previous suggestions for a safe zone of 2 cm in the posterior glenoid neck at the level of the supraglenoid tubercle [37]. In another study, injuries to the suprascapular nerve of up to 6% have been reported during surgery for shoulder instability, such as rotator cuff repair, [39, 40]. The median distance between glenoid and suprascapular nerve in the spinoglenoid region, measured on anatomic shoulder specimens, was 12 mm (range 6–15 mm) and 19 mm (range 11–23 mm) depending on shoulder rotation [34].

Even less data are available with regard to safe screw placement for (reverse) total shoulder arthroplasty [7], and only a few and methodologically divergent studies have used human cadavers to address the issue of safe screw placement for total or reverse shoulder arthroplasty so far [16, 17, 19, 39, 41, 42].

Furthermore, whilst a few anatomical studies have performed drill hole experiments, they did not take into account the sex dimorphism of the scapula [17, 19, 42], particularly as many patients undergoing total shoulder arthroplasty are women [4, 43]. In another study, morphological and radiological data were combined with drill hole experiments for screw placement, however the study was based on a historical bone collection, which may not accurately represent the anatomy of patients being treated today [41]. Thus, in the present study, we provide further data on the anatomical dimensions and inclination of the glenoid using an elderly cohort of body donors with a mixed sex ratio representative of patients undergoing total shoulder arthroplasty.

In our study we hypothesized that glenoid height and width might predict the distance to the scapular notch to facilitate and increase the safety of intraoperative drill hole placement. To allow comparison of the morphological measurements with routine preoperative treatment planning, 3D-CT measurements of version and inclination angles were additionally performed. To the best of our knowledge, this is one of the first studies using such a comprehensive approach.

## Methods

### Design of the study

Glenoid height and width were determined in a cadaver dissection study searching for potential predictors for the distance from the glenoid to the scapular notch. To gather further information relevant to anatomical and reverse total arthroplasty, the glenoid angles were also measured. To allow comparison with published data, which are predominantly recorded on isolated bones from anthropological and forensic collections (e.g., [26, 44]), the scapulae were subsequently extracted and orientated in a fixation device (similar to a previous method, [26]). In the present study, both anthropologists and anatomists measured glenoid angles and additional distances to broaden the database in the published literature. A radiologist experienced in orthopaedics performed 3D-CT measurements of version and inclination angles using the method of Friedman [45].

### Body donors

The study was performed on formalin-embalmed cadavers from the institutional body donation programme at the University of Zurich (http://www.anatomy.uzh.ch/de/koerperspende.html) subsequent to the 2nd year curricular dissection course for medical bachelor students. Prior to dissection, cadavers were routinely CT-scanned at the Institute of Forensic Medicine for teaching purposes, as is increasingly becoming established practice in anatomy courses (e.g., [46]). The study was approved by the Zurich Cantonal Ethics Committee (KEK ZH-Nr. 2014–0303). The study included the thoraces from 11 female and 7 male body donors, with an average age of 84 years (range: 60–98 years). All body donors gave informed consent for research, which the authors herewith gratefully acknowledge.

### Cadaver dissection and in situ measurements of distances

**Fig. 1:**
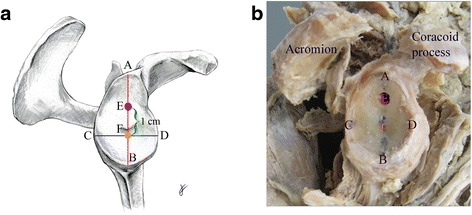
Schematic **a** and photographic **b** depictions of landmarks for measurements in a right scapula, lateral view. *A* Most cranial point of the glenoid cavity. *B* Most caudal point of the glenoid cavity. *C* Most dorsal point of the glenoid rim. *D* Most ventral point of the glenoid rim. *E* Drill hole entry. *F* Central peg hole

**Fig. 2:**
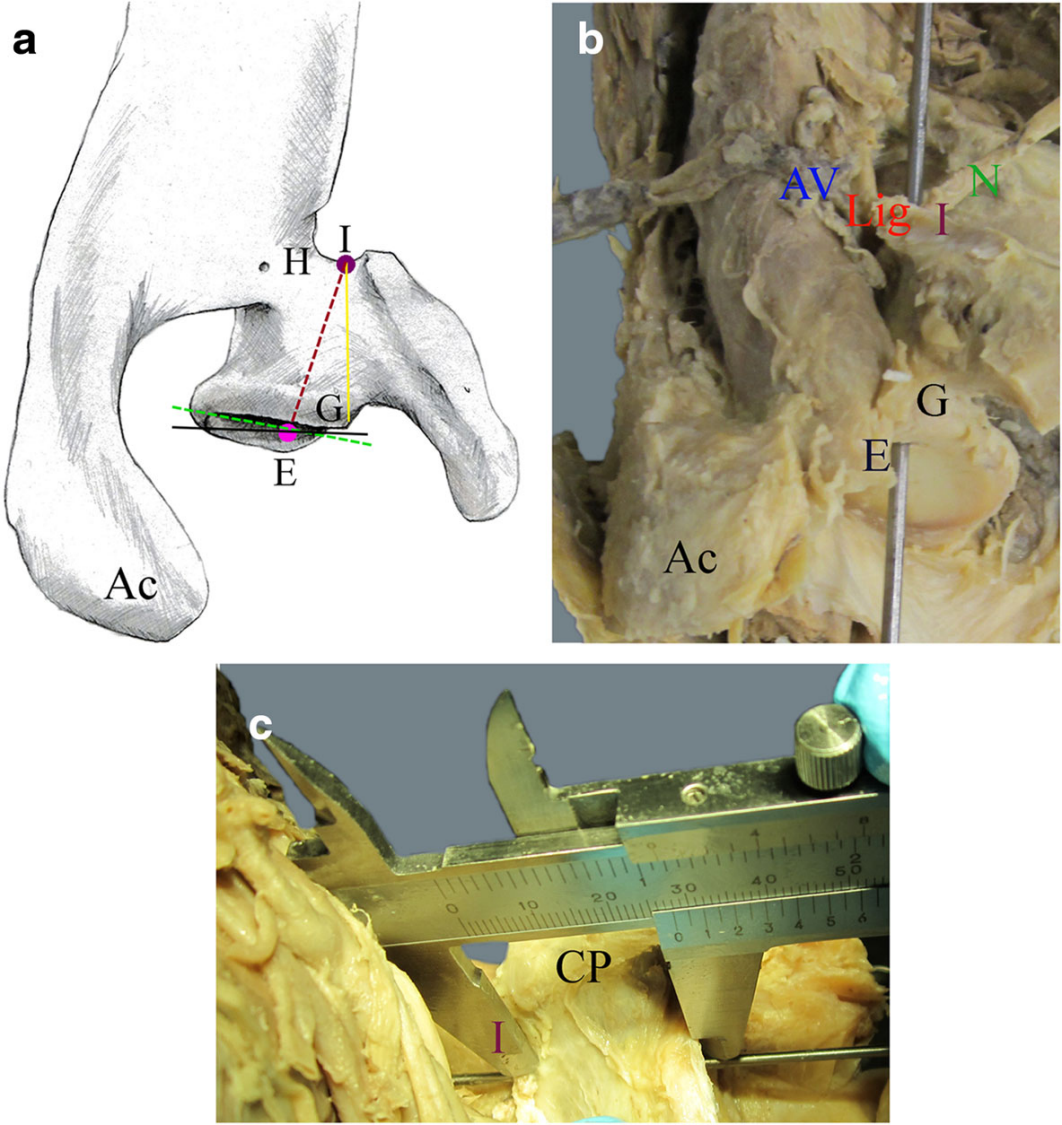
Schematic **a** and photographic **b** depictions of landmarks for measurements in a right scapula from a cranial view. **a** *Dotted violet line* designates the drilling canal. **b** Drill hole as depicted with a probe. **c** Photograph from a ventral view showing drill hole measurements using a calliper. *E* Drill hole entry. *I* Drill hole exit. *G* Supraglenoid tubercle. *Ac* Acromion. *CP* Coracoid process. *N* Suprascapular nerve. *AV* Suprascapular artery and vein. *Lig* Superior transverse scapular ligament. *Green line* designates the ante-/retroversion angle perpendicular to the glenoid inclination (*black line*)

After exposure of the glenohumeral joint by the delto-pectoral approach, maximal length and width of the glenoid were determined after Martin and Saller [47] as is commonly used intraoperatively (see: Fig. 1). In brief, a line (height) was drawn from the most cranial point of the glenoid cavity (Point A, Fig. 1) to the most caudal point (Point B, Fig. 1), and another line (width) from the most dorsal point of the glenoid rim (Point C, Fig. 1) to the most ventral point (Point D, Fig. 1). In a next step, the metal back glenoid L1 (SMR reverse shoulder prosthesis Systema Multiplana Randelli, provided by Lima Corporate SA, Italy) was selected according to the glenoid size (Small-R: *n* = 14 in 7 female donors, Standard: *n* = 22 in 11 donors, 7 males, 4 females). The central peg hole (Point F, Fig. 1) was designated at the appropriate position according to the recommendations by the manufacturer [48] for clinical practice. During surgery, special instruments are used to determine the correct position of the central K-wire, which is positioned before drilling the central peg hole of the metal back after preoperative determination of the center of the glenoid by CT scan. By analogy in our study, the intersection of the two lines (height and width) was constructed to determine the position of the central peg hole (Point F, Fig. 1). For the baseplate superior screw drilling procedure, the corresponding position of the superior screw entry point was designated 1 cm above the central peg hole at the 12-o’clock position (Point E, Fig. 1) for use as the origin of the drilling canal for the superior screw towards the suprascapular notch (Point H, Fig. 2a). All drillings were performed by the same two investigators (K.L, M.B.) according to instructions of an experienced shoulder surgeon (H.R.B.). Relevant distances between the glenoid and the scapular notch (see Fig. 2a,b) as well as the distance of the supraglenoid tubercle to the screw entry (Fig. 1a,b) were recorded with a calliper (Fig. 2c) to the nearest 1 mm. All measurements were taken repeatedly and recorded by the same three investigators (K.L., M.B., S.M.) as described [49]. The suprascapular notch was dissected to identify the suprascapular nerve and blood vessels (Fig. 1b).

### Measurements of glenoid angles on isolated scapular bones

**Fig. 3:**
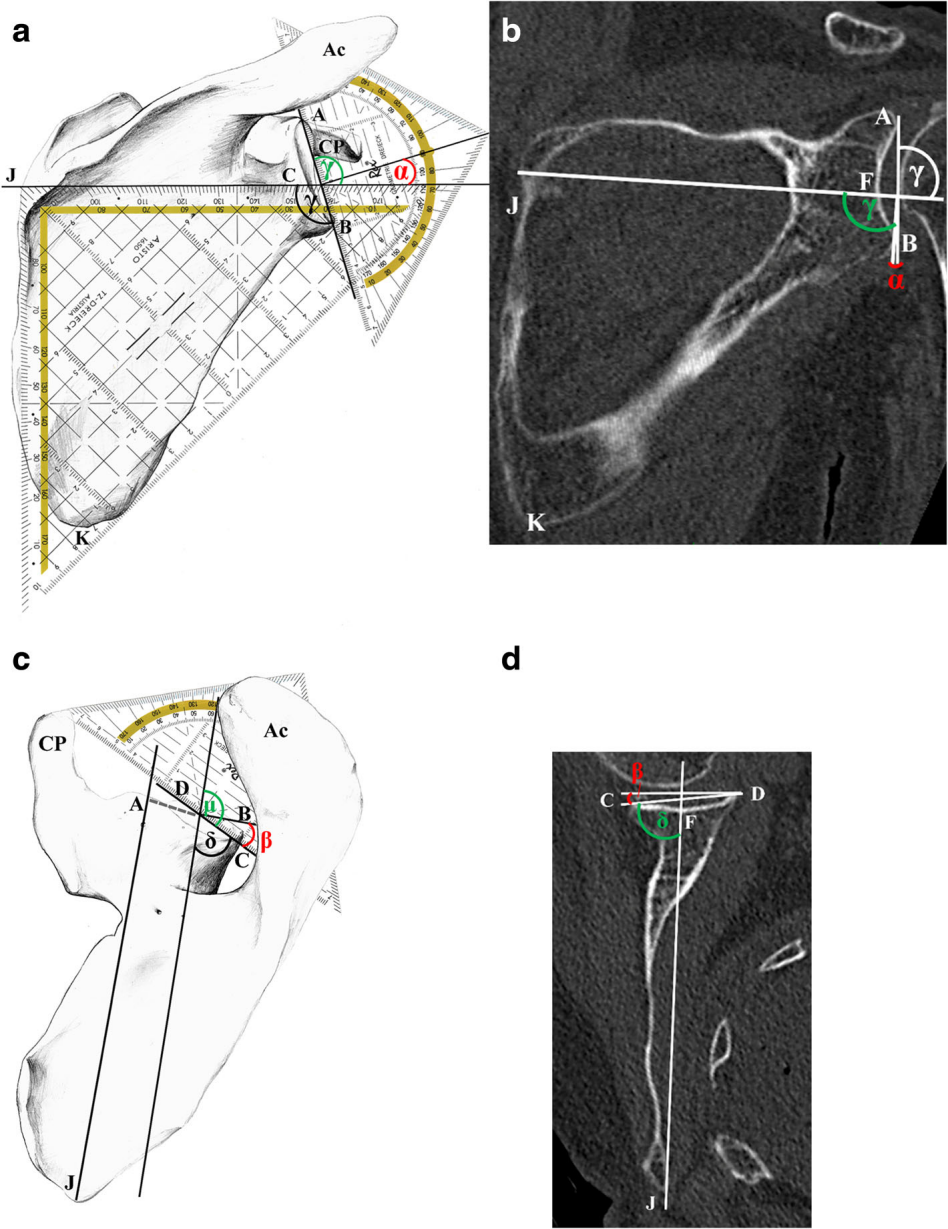
Measurements of glenoid angles using bones (**a**, **c**) and 3D-CT-reconstructions (**b**, **d**). **a** For determination of the inclination angle α, a geometrical triangle was adjusted on the dorsal side below the spine running from root of the spine (*J*) to the most posterior point at the glenoid rim (*C*). Another geometrical triangle was placed running from the supraglenoid (*A*) to the infraglenoid (*B*) tubercle. The angle γ (*green*) between these 2 axes was measured and from this, the inclination angle α (*red*) calculated by the formula: α = γ - 90°. **b** On the CT scan, the inclination angle α was determined in the coronal plane. *A* line was drawn from the root of the spine (J) to the midpoint of the glenoid cavity (*F*). A second line was drawn through the most cranial (*A*) and caudal (*B*) points of the glenoid rim. Angle γ (*green*) was measured between the axes of these two lines in the caudal direction. From this, the glenoid angle α (*red*) was calculated by the formula α = γ - 90°. **c** A geometrical triangle was used to designate the intersecting point between the perpendicular starting at *A* (extrapolation of the dotted line) to the middle of the line between *C* to *D*. From this, the angle μ (*green*) was measured between the axes of these two lines. In a next step, the angle δ was calculated by the formula: δ = 180°- μ and then the ante-/retroversion angle β (*red*) was converted by the formula: β = δ - 90°. **d** On the CT scan, the ante-/retroversion angle β was determined in the transversal plane according to Friedman [45]. A line was drawn through the root of the spine (*J*) to the midpoint of the glenoid cavity (*F*). A second line was drawn through the most posterior (*C*) and anterior (*D*) points of the glenoid rim. Angle δ (*green*) was measured between the axes of these two lines in the sagittal direction and from this, angle β (*red*) was calculated by the formula: β = δ - 90°. *Ac* Acromion, *CP* coracoid process

After disarticulation, extraction and removal of soft tissue, the scapulae were orientated in a fixation device. The inclination angle α (Fig. 3a) and the ante-/retroversion angle β (Fig. 3b) were measured by experienced anthropologists (S.M., N.F.) assisted by an experienced gross anatomist (K.L.) and the average result was taken as described previously [50]. In brief, a geometrical triangle was adjusted on the dorsal side below the spine from the root of the spine (J, see Fig. 3a) to the most posterior point at the glenoid rim (C). Another geometrical triangle was placed from the supraglenoid (A) to the infraglenoid (B) tubercle. The angle γ between these 2 axes was measured (Fig. 3a) and from this, the inclination angle α was calculated by the formula: α = γ - 90°. The ante-/retroversion angle β (Fig. 3c) was determined perpendicular to glenoid inclination, and the angles were recorded as (−) for retroversion and (+) for anteversion similar to 3D-CT-reconstructed measurements [51]. A probe was adjusted from root of the spine (J) along the supraspinous fossa towards the supraglenoid tubercle (A). A geometrical triangle was used to designate the intersecting point between the perpendiculars from the supraglenoid tubercle (A) to the middle of the line traversing the glenoid (C to D). From this, the angle μ was measured between the axes of these two lines. To allow comparison with the CT scan, the ante-/retroversion angle β had to be calculated: in a first step, the angle δ was calculated by the formula: δ = 180°- μ, and in a next step, the ante-/retroversion angle β was converted by the formula: β = δ - 90°.

### Measurements of glenoid angles by CT scan analysis

The CT scans of the cadavers were performed on a 128-slice CT scanner (Somatom Definition Flash, Siemens Medical Solutions, Forchheim, Germany). The reconstruction parameters were 120 kV, 500 reference mAs dose modulation (CAREdose4D™, Siemens Medical Solutions), and 0.6 mm slice thickness with 0.4 mm increments with a hard kernel in the osseous windows. Reconstructions were made in a small field of view adapted to the shoulder. The DICOM files were transferred to OSIRIX™ (Pixmeo SARL, Bernex, Switzerland). In the 3D multiplanar reconstruction mode, a plane of the scapula was defined by the midpoint of the root of the scapular spine (J, Fig. 3b), the centre of the glenoid (F) and the most distal point of the inferior scapula angle (K) as previously described [52]. Measurements were performed by a radiologist experienced in orthopaedics (F.M.B.) using established techniques [15, 53].

The inclination angle α was determined in the coronal plane (Fig. 3b). In brief, a line was drawn through the root of the scapular spine (J) to the midpoint of the glenoid cavity (F). A second line was drawn through the most cranial (A, see Fig. 3b) and caudal (B) points of the glenoid rim. Angle γ was measured between the axes of these two lines in the caudal direction. For comparison of the CT data with the angle α in the extracted scapulae (Fig. 3a) and with published data, the angle γ was converted by the formula α = γ - 90°.

The ante-/retroversion angle β was determined in the transversal plane (Fig. 3d) according to the method by Friedman et al. [45]. In brief, a line was drawn through the root of the scapular spine (J) to the midpoint of the glenoid cavity (F). A second line was drawn through the most posterior (C) and anterior (D) points of the glenoid rim. Angle δ was measured between the axes of these two lines in the sagittal direction (Fig. 3d). For comparison with the angle β in the extracted scapulae (Fig. 3c) and published data, the angle β had to be calculated by the formula: β = δ - 90°. Negative values indicate retroversion, positive values indicate an anteversion of the glenoid.

### Statistics

For statistical calculations, Microsoft Excel and IBM SPSS 23 were used. Data were examined by Shapiro-Wilk test to check for normal distribution and calculating Pearson's correlation coefficient (r). Significances were calculated with 2-tailed Student’s *t*-test. A significance level *p* < 0.05 was considered statistically significant.

## Results

### Measurements of the glenoid cavity

For both, male and female samples combined (Table 1), the average maximum height of the glenoid (A-B, Fig. 1a,b) was 36.6 mm ± 3.6 mm (range 31.0-43.6 mm), and width (C-D, Fig. 1a,b) was 27.8 mm ± 3.1 mm (range 23.5-34.7 mm) with a significant sex dimorphism (p ≤ 0.001). In males (Table 2), the glenoid height was 39.5 mm ± 3.5 mm (range 33.5-43.6 mm) and width 30.3 mm ± 3.3 mm (range 24.5-34.7 mm), and in females (Table 2), the glenoid height was 34.8 mm ± 2.2 mm (range 31.0-38.3 mm) and width 26.2 mm ± 1.6 mm (range 23.5-29.3 mm). Glenoid height and width correlated with each other (correlation r = 0.90).

**Table 1:** Measurements in cadaveric specimens from male and female donors combined

Measurement	Landmarks (from-to)	Average (mm)	SD (mm)	Range (mm)
Glenoid height	A-B	36.6	3.6	31.0-43.6
Glenoid width	C-D	27.8	3.1	23.5-34.7
Supraglenoid tubercle to superior screw (entrance)	G-E	13.2	3.4	7.0-20.3
Supraglenoid tubercle to scapular notch (centre caudal)	G-H	32.7	2.8	26.0-39.0
Superior screw entrance and exit	E-I	27.2	6.0	15.0-37.5
Supraglenoid tubercle to superior screw exit in suprascapular notch	G-I	26.8	6.5	14.0-37.0

*SD* standard deviation

**Table 2:** Measurements of scapulae and scapula angles sorted by sex

	Sex	N	Mean (mm)	SD (mm)	Range (mm)
Glenoid height***	m	14	39.5	3.5	33.5-43.6
	f	22	34.8	2.2	31.0-38.3
Glenoid width***	m	14	30.3	3.3	24.5-34.7
	f	22	26.2	1.6	23.5-29.3
Supraglenoid tubercle to superior screw (entrance)*	m	14	14.9	3.6	8.0-20.3
	f	22	12.1	2.8	7.0-17.1
Supraglenoid tubercle to scapular notch (centre caudal)*	m	14	33.9	3.2	27.5-39.0
	f	22	32.0	2.3	26.0-35.0
Superior screw entrance and exit	m	14	29.4	5.7	18.4-37.5
	f	22	25.8	5.9	15.0-33.9
Supraglenoid tubercle to superior screw exit in suprascapular notch*	m	14	29.6	5.9	19.3-37.0
	f	22	25.0	6.3	14.0-32.8
Inclination CT	m	14	15.0	8.0	−3-26.0
	f	22	12.0	6.0	1.0-26.0
Inclination scapular bone	m	14	5.0	3.0	1.5-13.5
	f	22	4.0	3.0	0.5-9.0
Ante-/retroversion angle CT	m	14	0	3.0	−5.0-6.0
	f	22	−1.0	5.0	−10.0-10.0
Ante-/retroversion angle scapular bone	m	14	−3,5	4.5	−13.5-3.5
	f	22	−4.0	4.0	−10.5-3.5

*SD* standard deviation

***indicates a *P* value < 0.001

**indicates a *P* value < 0.01

*indicates a *P* value < 0.05 as statistically calculated with *t*-test and Pearson correlation

### Measurement of the distances

For both, male and female samples combined, the length of the drilling canal (E-I, Fig. 2a, b) measured externally via calliper (Fig. 2c) was 27.2 mm ± 6.0 mm (range 15.0-37.5 mm). In detail, in males, the distance from the entry point of the superior screw to its exit in the scapular notch (E-I, Fig. 2a, b) was 29.4 mm ± 5.7 mm (range 18.4-37.5 mm), and in females 25.8 mm ± 5.9 mm (range 15.0-33.9 mm) however, these distances were not significantly different from each other. The distance from the supraglenoid tubercle to the superior screw exit in the scapular notch (G-I, Fig. 2a, b) was 26.8 mm ± 6.5 mm (range 14.0-37.0 mm) with a significant sex difference (*p* < 0.05). In detail, in males, the distance from the supraglenoid tubercle to the screw exit in the scapular notch (E-I, Fig. 2a, b) was 29.6 mm ± 5.9 mm (range 19.3-37.0 mm), and in females 25.0 mm ± 6.3 mm (range 14.0-32.8 mm). Further distances are summarized in Tables 1 and 2. The distance from the entry point of the superior screw to its exit in the scapular notch (E-I) did not significantly correlate with the glenoid height (correlation *r* = 0.31) and width (correlation *r* = 0.28).

## Measurements of glenoid angles

The average inclination angle (Fig. 3a, b) from male and female donors combined was 13.0° ± 7.0° (range −3.0° - 26.0°) as determined by 3D-CT (Fig. 3b). When measured on orientated fixed skeletal specimens (Fig. 3a), it was 5.0° ± 3.0° (range 0.5° - 13.5°). The average ante-/retroversion angle (Fig. 3c,d) from male and female donors combined was −1.0° ± 4.0° (range −10.0° - 10.0°) as determined by 3D-CT (Fig. 3d). When measured on orientated fixed skeletal specimens (Fig. 3c), it was −3.5° ± 4.0° (range −13.5 - 3.5). Data separated by sex are listed in Table 2. For measurements on the extracted scapulae, no significant interobserver error was found, but significant differences were observed between CT and morphological measurements: *p* ≤ 0.001 for inclination angles, and *p* ≤ 0.01 and *p* ≤ 0.001, respectively, for the ante-/retroversion angles.

## Discussion

In this multimodality approach, we measured the glenoid fossa and its distance from the scapular notch. A few studies on human cadavers have addressed the issue of safe screw placement for total shoulder arthroplasty so far (Table 3) using baseplates with 3 [41] or 4 screws [9, 17, 19, 42] with different orientations of the baseplate and drilling canal, respectively. Although 4 screw holes offer the advantage of good fixation, the required drilling procedures may cause further bone loss and thus weaken the construct even more [19, 41].

**Table 3:** Comparison of distances from the present study in male and female donors combined (for further details see Tables 1 and 2) with published data using drilling experiments on human cadaver specimens. Please note that the respective distances can be estimated only very roughly due to the different approaches of the published data. BP: baseplate

Study	Glenoid height (mm)	Glenoid width (mm)	Superior screw entrance and exit (drilling canal, mm)
Present study	36.6 (range 31–43.6)	27.8 (range 23.5-34.7)	27.2 (range 15–37.5)
DiStefano et al. [19]	39.5 ± 2.6	31 ± 2.5	Optimal screw length 35 ± 8 depending on angulation, inferior BP inclination
Molony et al. [17]	No data	No data	Screw length 36.6 (range 32–42)
Hart et al. [42]	No data	No data	29.3 (13–43) 1-o’clock position
Chae et al. [9]	25 mm BP: 32.6 ± 2.5 29 mm BP: 32.1 ± 2.4	25 mm BP: 23.3 ± 2.0 29 mm BP: 23.3 ± 1.7	25 mm BP: 32 ± 6.4 29 mm BP: 25.4 ± 5.8

In the present study, we used a 2-screw system and laid special emphasis on the superior screw. In all cases, we used standard or small glenosphere baseplates to determine the drilling hole. The origin of the drilling canal for the superior screw towards the suprascapular notch was designated at the 12-o’clock position in a centrally orientated peg glenosphere as recommended by the manufacturer. In our study, length of the drilling canal showed an average for both sexes combined of 27.2 mm (29.4 mm in males, 25.8 mm in females) with a calliper adjusted to the probe within the drilling canal (Fig. 2c). These results are shorter than reported by other groups (see Table 3), but resemble more the proposed optimal length of 29 mm (range 18–29 mm) for the ideal screw position as calculated from CT-simulations [41], and correlate for female to the published mean length of 25.4 mm in the female subgroup of 29 mm baseplate (see Table 3) in another study [9].

To allow comparison with other studies from different countries, we expanded our measurements to the distance from the supraglenoid tubercle to the center of the scapular notch. Our data from Switzerland had an average of 32.7 mm, which is similar to that of 31 mm from Italy [27], 33 mm from Turkey [54] and Germany [2] and of 30 mm [37] and 34.2 mm [39], from the USA. Slightly shorter distances of 28.7-29.1 mm were found in a study from India [32] and Kenya with a range of 27.3-30.1 mm depending on the scapular notch type [55] and of 29 mm (23–35 mm) from Japan [38].

In a study on the Latarjet procedure [2], distances of 33 mm (range 31–35 mm) between the suprascapular nerve and 3 reference points along the glenoid rim were measured, including the supraglenoid tubercle, of which the latter could be used for comparison with our average of 32.7 mm. Nevertheless, it has to be noted that in our investigated Middle European population, the distance between screw entry and suprascapular notch was little as 15 mm in females. To the best of our knowledge a similar short distance of 13 mm has only been previously reported in a single study [42], but without providing further data regarding the specimens used. Also a recent clinical study has emphasised the increasing use of small baseplates [56]. This would mean that the proposed safe distance of 20 mm described above might entail a potential risk in some smaller individuals, or individuals from other populations, or particularly in women. Thus, more studies are needed to investigate the sex dimorphism of scapular size in males and females.

Integration of CT and/or MRI scans in planning shoulder arthroplasty is therefore generally recommended.

In the present study, glenoid size showed an average height of 39.5 mm and width of 30.3 mm for males, and height of 34.8 mm and width of 26.2 mm for females. We had hypothesized that glenoid height and width should correlate with each other, which we herewith confirm. However, no correlation was found between glenoid height or width, and the length of the drilling canal towards the scapular notch.

The glenoids measured in the present study affirm the described sex difference [25]. Similar data from Middle Europe showed a glenoid height of 40 mm and width of 29 mm for males and height of 36.1 mm and width of 25.7 mm for females [57] as well as height of 31.7 ± 3.7 mm and width of 24.7 ± 3.5 mm for both sexes combined [58]. However, our data on glenoid size was smaller than the glenoid height of 45.7 mm in a USA study on 4 men and 4 women [59]. On the other hand, our results are larger than in men with a height of 37.5 mm and width of 27.8 mm, and in women, a height of 32.6 mm and width of 23.6 mm. The smaller data from this historical bone collection as compared to more recent data may support the assumption that this may not reflect the patients undergoing surgery today [26]. For a contemporary Mexican population, the need for better knowledge on sex dimorphism of glenoid size and the importance of population-specific discriminants for scapulae was highlighted for forensic scenes [44]. Further, a recent study postulated that larger sample sizes for ethnic groups should be explored. This study identified sex as the strongest independent predictor of glenoid size. Men exhibited a larger glenoid, however, patient height was found to be predictive only in patients of the same sex. The authors further observed variations in glenoid size and version also among ethnicities [58].

In the present study, on isolated scapulae a mean glenoid inclination angle of 5° in males and 4° in females were measured, which are rather similar to findings of 4° in male donors (range −7° - 15.8°), and 4.5° (range −1.5° - 15.3°) in female donors [26] and of 7.1° ± 1.7° in 4 men and 4 women [59]. In another study, a mean inclination angle of 12° (range −21° - 50°) was determined by postoperative CT-scanning of predominantly female patients, from which the authors concluded there was frequent malpositioning of the baseplate [6]. Nevertheless, the mean value of 13° for the inclination angle measured by CT was higher as compared to our findings in bone, and to the CT-data of 1° in male and 4° in female normal glenoids from another study, which reported no significant difference to osteoarthritic type B2 glenoids [51].

Using historical scapulae with a mean age of 25.6 years, Churchill and co-workers [26] measured mean glenoid version angles of 0.35° in black and 3.49° in white men, and 0.79° in black and 2.8° in white women. Recently, using CT scans, a mean version of 0.05 ± 9.05 was measured from which the authors proposed that males are expected to exhibit 8.4° more retroversion than females, and Hispanics demonstrate 6.4° more anteversion compared to African-Americans [58]. Retroversion of 4° - 8° has been described as normal, while higher retroversion angles predispose for dorsal shoulder luxation and glenoid loosening secondary to abnormal forces across the implant and the cement-bone interface [12, 30].

We attribute our own retroversion angles of −3.5° on bones (range −13.5°- 4.5°) and of −1° on CT scans (range −10° - 10°) to the pronouncedly higher age of the population we investigated. Our data were comparatively similar to findings of −4° (range – 18° - 5°) measured in a stable control group of a clinical study [14] by MRI according to Friedman [45] and of - 8.5° ± 5.2° by 3D-CT-reconstruction [19]. In a study comparing a new 2D measurement method, retroversion angle of −19° ± 3° were measured in the control group, whilst the method by Friedman [45] revealed a retroversion of −1° ± 6° [13]. We attribute the discrepancies between bone and CT-measurements in our study to the complex morphology of the scapula, which in a minority of cases was difficult to orientate due to pronounced deformations in 6 samples from 4 individuals aged 81–94 years. Furthermore, remnants of cartilage present on the extracted scapulae may account for these observed differences. Nevertheless, the difference between our morphological and radiological measurements was within a similar order of magnitude as reported for 2D- and 3D-CT measurements, with a range for the glenoid version angle of 0.1°–23°, and for the inclination angle of 0.2°–4.5° [12–15, 52, 60, 61].

To summarise, proper sizing and correct placement of prosthetic components are mandatory. 3D-imaging and patient specific instrumentation will have to be based on a profound knowledge of glenoid morphology [8, 61]. We hereby add a new systematic dataset regarding glenoid size, version and inclination angles and scapular distances to the literature. The glenoid angles measured by morphological and 3D-CT reflect the differences in elderly male and female patients undergoing total shoulder arthroplasty.

However, there are some limitations in this study: First of all, measurements by 3D-CT differed from those on the isolated scapulae, which may, as outlined above, be expected from published differences in the literature. Secondly, drillings were performed using a 2-screw system with a clear focus on the superior screw and the danger to the suprascapular nerve. Thirdly, central positioning of the baseplate was applied as used in previous work [17, 42], but differed for another study [19], which used inferior inclination of the baseplate. Nevertheless, to the best of our knowledge, this is one of the first studies to comprehensively present such a broad spectrum of morphological data in a mixed elderly population.

## Conclusions

As also indicated in the literature, in some women and smaller individuals, smaller baseplates should be selected. The published safe zone of 20 mm is generally feasible also for superior screw placement. However, in small patients careful consideration is necessary that the distance between the glenoid and the scapular notch may be shorter than expected and be as little as 13 and 15 mm, respectively. While glenoid height and width correlated with each other in the present data set, no correlation between glenoid height or width with the length of the drilling canal towards the scapular notch was found. Preoperative CT-based treatment planning to determine version and inclination angles as well as the distance between the glenoid and the scapular notch is recommended.

## Abbreviations

RTSA: Reverse total shoulder arthroplasty
TSA: Anatomical total shoulder arthroplasty
